# Intestinal Dysbiosis and Tryptophan Metabolism in Autoimmunity

**DOI:** 10.3389/fimmu.2020.01741

**Published:** 2020-08-04

**Authors:** Josephine Brown, Brian Robusto, Laurence Morel

**Affiliations:** Department of Pathology, Immunology, and Laboratory Medicine, University of Florida, Gainesville, FL, United States

**Keywords:** autoimmunity, microbiota, dysbiosis, metabolites, tryptophan, kynurenine

## Abstract

The development of autoimmunity involves complex interactions between genetics and environmental triggers. The gut microbiota is an important environmental constituent that can heavily influence both local and systemic immune reactivity through distinct mechanisms. It is therefore a relevant environmental trigger or amplifier to consider in autoimmunity. This review will examine recent evidence for an association between intestinal dysbiosis and autoimmune diseases, and the mechanisms by which the gut microbiota may contribute to autoimmune activation. We will specifically focus on recent studies connecting tryptophan metabolism to autoimmune disease pathogenesis and discuss evidence for a microbial origin. This will be discussed in the context of our current understanding of how tryptophan metabolites regulate immune responses, and how it may, or may not, be applicable to autoimmunity.

## Introduction

Interactions between host genetics and environmental triggers are known to underlie the development of autoimmune disorders. Among these environmental triggers, intricate relationships between the host and its microbiota have a large impact on health and disease. In particular, the understanding of host-microbiota relationships has generated an intense interest in autoimmunity. The symbiotic relationship between the host and microbiota is bolstered by mechanisms to maintain homeostasis between the two entities, including the microbiota-mediated regulation and maturation of the immune system (1, 2). Commensals and their products contribute to the integrity of the intestinal barrier (3) and promote immunological tolerance (4, 5). Therefore, pathogenic alterations to gut microbial communities, or intestinal dysbiosis, may compromise the capacity of the microbiota to limit inflammation, which may be especially deleterious in genetically susceptible hosts where endogenous mechanisms to control inflammation are already impaired.

Intestinal dysbiosis has been documented in human and murine autoimmunity (6–21). Specific classes of microbes that may be associated with disease have been identified (6, 9, 17, 22), but causal links between specific bacteria and autoimmune manifestations are still rare. It is not clear if host genetics, environmental conditions, or interactions between them, play a role in establishing disrupted microbial communities. In addition, whether or not dysbiosis plays a role in disease initiation or amplification remains to be elucidated. There are three main mechanisms by which the microbiota could play a role in autoimmunity: molecular mimicry (23–25), an impaired intestinal barrier that may promote bacterial translocation (20, 26), and an altered abundance of microbial metabolites with immunoregulatory functions (21). Each of these mechanisms has the potential to promote inflammation and consequent tissue damage, especially when combined with genetic susceptibility to autoimmune diseases.

Molecular mimicry has long been considered to be a major mechanism leading to autoimmunity (27). In this process, microbial antigens possess high homology to host antigens, leading to cross-reactive immune responses and chronic inflammation. There is evidence for pathogen-induced molecular mimicry in autoimmunity (27). For example, *Streptococcus pyogenes* is a trigger of rheumatic fever (28). Recently, studies have also shown evidence for intestinal commensals eliciting cross-reactive immune responses with self-antigens (23–25).

The intestinal barrier simultaneously prevents immune responses against commensals and excludes pathogens (29). Any compromise to this barrier has the potential to elicit inflammatory responses against overabundant leaked microbial antigens. Additionally, bacteria could translocate across a compromised barrier and disseminate to distal organs to promote inflammatory responses (20, 26). Therefore, bacterial translocation may trigger or amplify inflammation.

**Figure 1:**
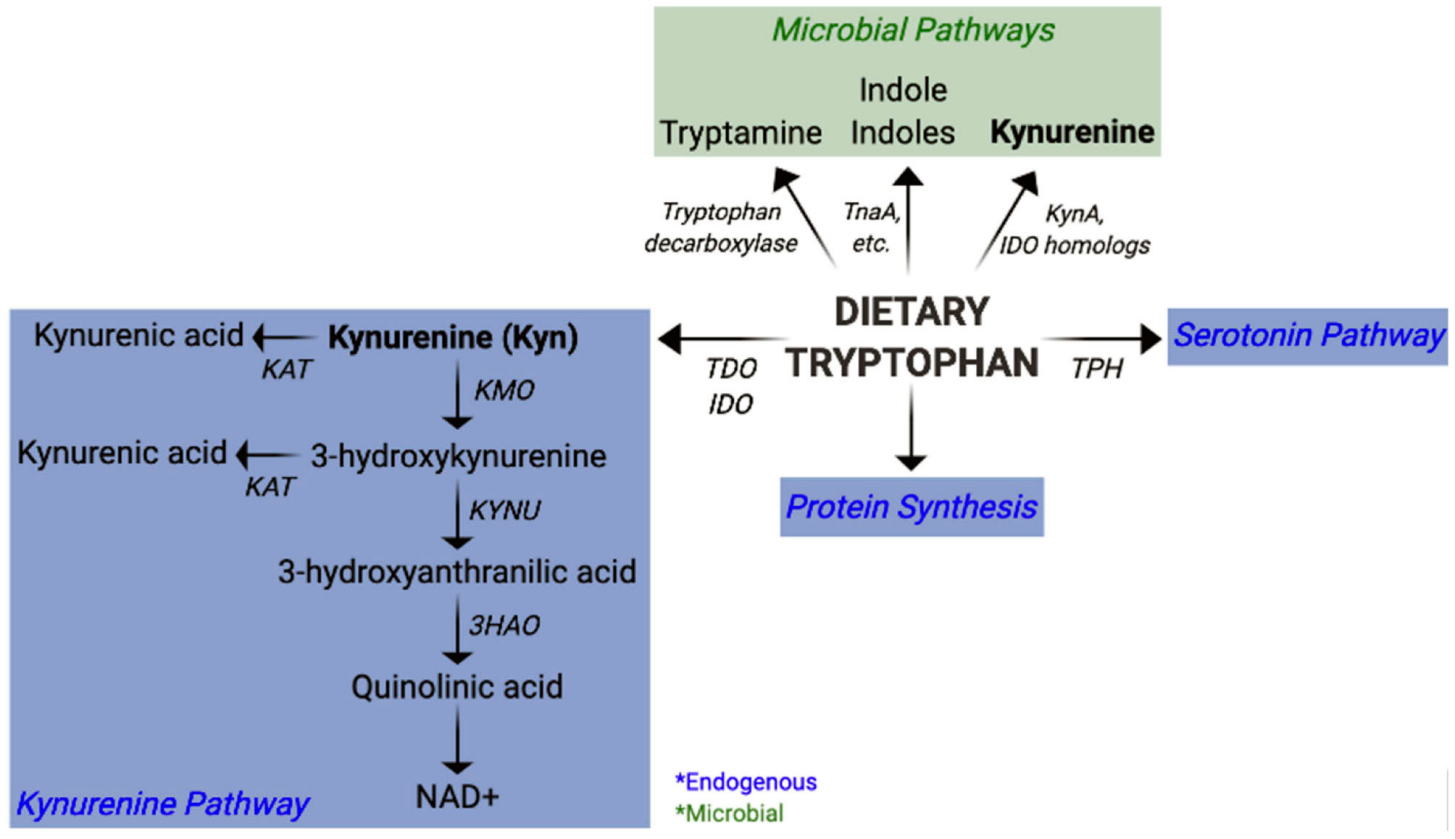
Pathways of mammalian and microbial tryptophan metabolism. Overview of host (endogenous) and bacterial pathways of tryptophan metabolism highlighting kynurenine synthesis.

In the past decade, it has become increasingly evident that microbial metabolites play an indispensable role in immune modulation and intestinal homeostasis (30–35). The short chain fatty acids (SCFAs), acetate, butyrate, and propionate, are derived from commensal fermentation of dietary fiber. Collectively, SCFAs promote intestinal homeostasis via their tolerogenic properties and their ability to reinforce intestinal barrier integrity (36). Furthermore, bile acids are metabolized by the microbiota into secondary bile acids that also have immune modulatory activity (37). An altered distribution of tryptophan (Trp) metabolites has been identified in numerous autoimmune diseases (38–49). Independently of endogenous host Trp metabolism, enzymes in the intestinal microbiota catabolize Trp to produce various metabolites (Figure 1) that play an important role in immune modulation and microbiota-host communication (50–53). The contribution of these metabolites to autoimmune diseases has, however, not been fully appreciated.

Here we review autoimmune-associated microbial dysbiosis and mechanisms by which it may contribute to pathogenesis. We focus on systemic lupus erythematosus (SLE), rheumatoid arthritis (RA), and multiple sclerosis (MS), due to the abundance of studies reporting association and investigating the mechanisms of microbial contributions. Among these, we highlight host and commensal-derived Trp metabolites as anti- and pro-inflammatory mediators in autoimmunity, illustrating complex interactions that need to be further investigated.

## Evidence for a Contribution of Intestinal Microbial Dysbiosis to Autoimmune Pathogenesis

### Systemic Lupus Erythematosus

SLE is a chronic autoimmune disease of complex etiology characterized by the presence of autoantibodies against cellular antigens, which form immune complexes that are responsible for multi-organ heterogeneous clinical manifestations (54). Several features emerge from studies that have compared the distribution of fecal bacterial 16S rDNA between SLE patients and healthy controls (HCs). A lower *Firmicutes/Bacteroidetes* (F/B) ratio has been found in two independent cohorts of SLE patients (7, 13), but this has not been confirmed in other cohorts (16, 25). A reduction in microbial diversity, commonly associated with dysbiosis and disease state, has been reported in the latter two studies with an inverse correlation between diversity and disease activity (25). SLE subjects also presented an expansion of specific phyla: *Prevotella* (7, 13), which has also been reported in RA patients (6, 15, 17), *Proteobacteria* (13, 16) and *Actinobacteria* (13). In a more detailed study, *Ruminococcus gnavus* and *Veillonella* spp. were highly enriched in SLE feces and the abundance of *R. gnavus* positively correlated with disease activity (25). Importantly, SLE patients with renal involvement had a greater abundance of *R. gnavus* (25). Taken together, these data demonstrate a state of intestinal dysbiosis in SLE patients that may be associated with disease activity.

Lupus-prone NZB/W F1 mice present with intestinal dysbiosis at disease onset and exhibit an increased relative abundance of *Lactobacillus* in established disease compared to the pre-disease state (16). Administration of dexamethasone, a common treatment in SLE patients, which also attenuates disease in NZB/W F1 mice (55), decreased the relative abundance of *Lactobacillus* and increased microbial diversity (16), suggesting that some *Lactobacillus* spp. are associated with disease in this model. However, colonization of NZB/W F1 mice with *L. paracasei* reduced disease-associated cardiac complications (56), although the effects on autoimmune manifestations were not examined. Moreover, treatment of NZB/W F1 mice with the probiotic strain *L. fermentum* changed their microbiota and reduced the F/B ratio (57), improved gut barrier, and endothelial integrity, as well as decreased serum anti-dsDNA IgG (57). Collectively, these studies suggest that an outgrowth of *Lactobacillus* may be associated with disease in the NZB/W F1 model. On the other hand, specific *Lactobacillus* probiotic strains improve disease manifestations. It is therefore possible that specific strains of *Lactobacillus* are therapeutic, while others exacerbate autoimmune complications in NZB/W F1 mice via unknown mechanisms.

MRL/lpr lupus-prone mice also present an intestinal dysbiosis, but it is characterized by a higher microbial diversity, decreased *Lactobacillaceae*, and increased *Lachnospiraceae, Rikenellaceae*, and *Ruminococcaceae* (8) compared to non-autoimmune controls. However, specific pathogen free (SPF) and germ free (GF) MRL/lpr mice presented similar disease manifestations (58), indicating that the microbiota is not required for disease initiation and development. Disease was attenuated in this strain by a broad-spectrum antibiotic cocktail or by vancomycin alone, which decreased the abundance of *Bacteroidales* and *Clostridiales* as well as increased the abundance of *Lactobacillus* (59). This suggests that dysbiosis amplifies established disease and that *Lactobacillus* spp. are protective in this model. Additionally, MRL/lpr mice have a compromised intestinal barrier leading to gut leakage, and colonization with *Lactobacillus* spp. improved intestinal barrier integrity, and improved disease outcomes (16). In summary, these data suggest that a low abundance of *Lactobacillus* may be associated with autoimmune manifestations in MRL/lpr mice by promoting leaky gut and potentially bacterial translocation.

Intestinal dysbiosis has also been shown in the B6.*Sle1.Sle2.Sle3* triple congenic (TC) spontaneous model of lupus (18, 21). A unique advantage of the TC model is the 95% genetic similarity between TC and non-autoimmune C57BL6 (B6) mice, thus allowing B6 to serve as a true non-autoimmune genetic control. No differences were observed in microbial diversity, but some taxa were enriched in autoantibody positive TC mice, including *Paraprevotellaceae, Paraprevotella, Lactobacillales, Lactobacillaceae*, and *Lactobacillus* (21). The increased abundance of *Prevotella* is consistent with the results obtained in SLE (7, 13) and RA patients (6, 15, 17), as well as the TRL7 transgenic model of lupus (20). Such differences were not observed in young TC mice before they produce autoantibodies (21), suggesting that gut dysbiosis develops with disease. Fecal microbiota transfers (FMT) from autoantibody positive TC mice induced the production of autoantibodies in GF B6 recipients, and increased the frequency of germinal center B cells, plasma cells, and follicular helper T cells (Tfh) (18, 21), all of which are phenotypes strongly associated with lupus. Interestingly, FMTs from either young TC mice or from TC.*Rag1*^−/−^ mice did not induce autoimmune phenotypes in GF B6 recipients (21), further indicating that the pro-inflammatory functions of TC microbiota occur after the development of autoimmunity and that they require the presence of lymphocytes. Furthermore, autoimmune phenotypes can be transferred horizontally between TC and B6 mice by co-housing (21). Together, these data demonstrate that microbial dysbiosis in the TC model amplifies autoimmune activation rather than driving disease initiation.

Exact mechanisms by which immune reactivity occurs against specific autoantigens in lupus remain elusive, but evidence suggests that molecular mimicry could be one of them. Autoantibodies against the RNA binding protein Ro60 are produced by a majority of lupus patients (60). Bacteria such as *Bacteroides thetaiotaomicron* that express orthologs of Ro60 have been identified in the intestinal microbiota of SLE patients and HCs with a similar abundance (24). However, a microbial origin of Ro60 autoreactivity was suggested when T cells isolated from anti-Ro60 positive SLE patients proliferated in response to microbial Ro60, and the sera from these patients bound microbial Ro60 orthologs (24). This hypothesis was verified when GF mice monocolonized with *B. thetaiotaomicron* produced anti-Ro60 antibodies (24). Molecular mimicry was also suggested when sera from SLE patients reacted with a *R. gnavus* lipoglycan, and there was a positive correlation between the serum levels of anti-lipoglycan antibodies and anti-dsDNA autoantibodies (25). Furthermore, patient anti-dsDNA IgG cross-reacted with *R. gnavus* antigens (25). These findings were confirmed in a separate cohort of SLE patients (25), suggesting that the association of *R. gnavus* expansion with disease activity and the cross-reactivity of *R. gnavus* antigens with mammalian DNA may be general to SLE. Overall, these studies emphasize the presence of microbial antigens to which SLE patients display immune reactivity, suggesting that certain bacteria and their products may play a role in pathogenesis through molecular mimicry.

SLE patients present signs of leaky gut, such as increased fecal IgM and IgG (25), in addition to fecal calprotectin (25, 26), fecal albumin (26), and serum soluble CD14 (25). *Enterococcus gallinarum* DNA was detected in liver biopsies from SLE and autoimmune hepatitis patients (26). *E. gallinarum* increased the expression of autoimmune promoting factors such as beta-2 glycoprotein 1 (GPI) and type I interferon when cultured with primary human hepatocytes (26). Further, antibodies against *E. gallinarum*-specific RNA were detected in both groups of autoimmune subjects (26). This same study showed that a compromised intestinal barrier in (NZW × BXSB)F1 lupus-prone mice allows *E. gallinarum* to translocate to the mesenteric lymph node (mLN) and liver, where it activates the AhR pathway and promotes autoantibody production (26). Bacterial translocation was also demonstrated in the TLR7 transgenic model of lupus (20), in which *Lactobacilllus reuteri* translocates across the intestinal barrier to stimulate type I interferon production, and therefore exacerbate disease activity (20). The abundance of *L reuteri* and its translocation were reduced by a high fiber diet that alleviated autoimmune manifestations (20). Together, these data indicate an impaired intestinal barrier may allow bacteria to gain entry to circulation and distal organs in SLE patients and mouse models of the disease. However, systemic autoimmunity develops in the TC model in the absence of leaky gut (21). In this model, in which neither molecular mimicry nor leaky gut seem to be the prevalent mechanism, the dysbiotic microbiota may amplify disease through metabolites, which will be discussed later in this review. The differences between mouse models may reflect differences in genetic backgrounds, disease etiology, or disease severity, since the two “leaky gut” strains, MRL.lpr and (NZW × BXSB)F1, present an accelerated severe pathology as compared to TC mice. Additional screenings of other spontaneous or induced models should be performed to address these issues.

### Rheumatoid Arthritis

RA is characterized by prolonged synovial inflammation leading to bone erosion and cartilage destruction. Several studies have reported an altered intestinal microbial composition in RA patients (6, 10, 12, 15, 17, 19). A reduced microbial diversity in two cohorts was inversely correlated with disease duration (12, 19), while increased diversity positively correlated with treatment (12). This suggests that a decreased intestinal microbial diversity represents, at least, a biomarker and maybe a contributor to disease activity in RA. Alterations described in microbial taxa are not consistent between RA patient cohorts, which may be due to differences in ethnicity, sex, age, or environment. Intriguingly, however, expansions of *Prevotella* spp. have been consistently reported in RA patients (6, 15, 17). Increased abundance of *P. copri* was associated with disease in new-onset RA patients (6, 15) and with a higher risk for developing RA (17), suggesting that this microbe may play a role in disease initiation.

Evidence for a leaky gut in RA is not concrete, but it has been proposed that gut microbes could be internalized by immune cells in the gut, which may then traffic to the joints. Oral bacteria found in periodontitis may also translocate to inflamed joints where they can produce citrullinated peptides as well as promote inflammatory cytokines (61). DNA from *Prevotella* (62) and other bacterial species (63) has been identified in synovial fluid from RA patients. There, molecular mimicry is a likely mechanism for bacterial contribution to RA pathogenesis, as sequence homology exists between autoantigens and *Prevotella* antigens (23). An HLA-DR-presented *P. copri* peptide was found in PBMCs from recent onset RA patients, and it stimulated Th1 responses (62). These patients also had antibody responses against *P. copri* (62). Colonization of SKG mice, a spontaneous model of RA, with the microbiota from new-onset RA patients with increased *P. copri*, expanded intestinal Th17 cells and their responses to an RA autoantigen (15). SCFAs have been hypothesized to play a protective role in RA. RA patients consuming a high fiber diet presented increased numbers of Treg cells, as well as decreased titers of autoantibodies and joint inflammation compared to RA subjects consuming lower fiber diets, presumably due to SCFAs produced from fiber (64). Indeed, SCFAs regulate bone homeostasis through osteoclast metabolism in mice (65), so it is possible that these metabolites could be beneficial to patients.

Murine studies have provided some mechanistic insights on microbial contributions to autoimmune arthritis. Under GF conditions, K/BxN mice presented attenuated disease manifestations, as well as reduced autoantibody titers and frequency of germinal centers (22). Mechanistically, these changes were linked to a loss of Th17 cells in the lamina propria in the absence of segmented filamentous bacteria (SFB) (22). SFB-colonized GF K/BxN mice developed arthritis, which was associated with an induction of Th17 cells and a subsequent increase in autoantibody titers (22). In addition to driving Th17 responses in K/BxN mice, SFB also induced Tfh differentiation in Peyer's patches and promoted Tfh cell migration to systemic sites where germinal center responses and autoantibody titers increased (66). These experiments demonstrated a causal link between specific bacteria, SFB, and autoimmune arthritis through the expansion of Th17 and Tfh cells.

Collagen-induced arthritis (CIA) mice that develop disease showed microbiota perturbations, including *Lactobacillus* expansions, compared to those that do not develop disease (67). FMT from CIA mice into GF recipients induced an arthritis phenotype (67), demonstrating causality of this dysbiosis. Butyrate prevented disease in CIA mice (68), suggesting that bacterial metabolites resulting from dietary interventions may be beneficial in RA. Overall, these studies demonstrated a contribution of the microbiota to autoimmune joint inflammation.

### Multiple Sclerosis

MS is a chronic central nervous system (CNS) disease characterized by aberrant immune responses against myelin autoantigens leading to axonal nerve damage. Human fecal microbiome studies have reported differences relative to HCs (9, 11, 14). The microbiota from patients with relapsing-remitting MS who were in remission was more similar to the microbiota from HCs compared to those with active disease, although there was overlap between the active and remission states (11). Although no global differences were found between the microbiota of MS-discordant twins, mice colonized with microbiota from the MS-twins developed experimental autoimmune encephalomyelitis (EAE) more frequently than those colonized with the microbiota from healthy twins (69). Moreover, splenocytes from recipients of the MS-twin microbiota produced less IL-10 after stimulation *in vitro* (69). These results suggest a contribution of gut microbiota to MS that is independent from genetic susceptibility.

Unlike RA and SLE patients, *Prevotella* spp. were less abundant in MS patients (9). However, a reduced *Clostridia* abundance was reported in MS patients compared to HCs (9). Interestingly, *Clostridia* spp. increase the differentiation and expansion of Treg cells (4, 70), which suggests a protective role. Moreover, the abundance of several taxa, including *Methanobrevibacter* and *Akkermansia*, was correlated with an increased expression of genes in T cell and monocyte innate signaling pathways in the PBMCs of MS patients (14), suggesting a causal link. MS patients also show signs of leaky gut (71, 72), which could allow for translocation of these bacteria and subsequent involvement in CNS autoimmune pathogenesis.

Antibiotic treatment was protective when administered before EAE induction in B6 mice (73) and before disease onset in spontaneous octospinal encephalomyelitis (OSE) mice, which presented a reduced frequency of IL-17-producing T cells (74). However, antibiotics had no effect on established disease in either OSE mice or a transgenic model of spontaneous relapsing remitting disease (74). These findings demonstrate that reducing the microbiota in murine models of MS was beneficial only preventively. Single probiotic bacteria have also shown preventive effects. *L. reuteri* attenuated EAE progression when given before induction, noted by reduced spinal cord immune cell infiltration, as well as the frequency and function of Th1 and Th17 cells (75). Protection from induced disease also occurred after colonization with *L. paracasei* (76). *Bacteroides fragilis* prevented EAE by increasing the frequency of Treg cells (77). Similarly, *Clostridium butyricum* attenuated EAE by decreasing the frequency of Th17 cells and expanding that of Treg cells (78). Overall, mouse studies suggest that the microbiota contributes to MS autoimmune pathogenesis by modulating effector T cells but has little effect once pathogenic T cells have been generated.

Although clinical and animal studies have established an association between microbial dysbiosis and autoimmunity, it is unknown how shifts in microbial communities arise as few studies have examined the temporal relationship between microbiota alterations and disease progression. Dysbiosis may arise before disease onset due to host genetics or environmental factors, and in this case, it may play a role in disease initiation. However, another possibility is that dysbiosis is secondary to disease development and/or autoimmune activation, in which it may amplify rather than trigger disease. Therefore, more studies are needed to understand how dysbiosis is established and at what point it becomes pathogenic. Due to the complexity of human microbiota studies, including cohort heterogeneity and immunosuppressive medications, which themselves could alter the microbiota, murine studies, including colonization of GF mice, will be indispensable, at least in a first stage, to conduct these mechanistic studies.

## Tryptophan Metabolism in Autoimmunity

### Host and Microbial Tryptophan Metabolism Pathways

The essential amino acid Trp is a precursor for the endogenous synthesis of Kyn and serotonin by the host enzymes (Figure 1). Indoleamine-2,3-dioxygenases (IDO1, IDO2), and tryptophan-2,3-dioxygenase (TDO) are the mammalian enzymes responsible for catalyzing the synthesis of Kyn from Trp. TDO expression is mostly restricted to the liver, whereas IDO1 is expressed in numerous tissues, most notably in immune cells and the intestinal epithelium. IDO2 also participates in Kyn synthesis, though with a lower activity compared to IDO1 (79). Overall, little is known about how IDO2 participates in host physiology. Kyn can be metabolized by several downstream enzymes to give rise to additional metabolites, collectively referred to as “kynurenines” (Figure 1). It has been estimated that about 90% of dietary Trp is metabolized through the Kyn pathway, largely by liver TDO (50). Additionally, hepatocytes express all enzymes within the Kyn pathway and represent a significant source for downstream kynurenines. Some kynurenines, such as quinolinic acid and kynurenic acid exhibit neuromodulatory properties and have thus been implicated in numerous peripheral and CNS diseases (80, 81). *De novo* synthesis of NAD, an essential co-factor in energy metabolism, represents the last step of the Kyn pathway in some cells, such as hepatocytes and macrophages (50, 82).

Bacteria also catabolize Trp to produce a plethora of bioactive metabolites (51–53). Some bacteria synthesize indole from Trp via the tryptophanase enzyme (TnaA), while others synthesize additional indoles such as indole lactic acid (ILA), indole propionic acid (IPA), and indole aldehyde (IAld) through separate pathways (51, 53). Furthermore, some bacteria use the tryptophan decarboxylase enzyme to generate tryptamine (83), which structurally resembles serotonin and binds to intestinal serotonin receptors to regulate intestinal transit (84). Gut microbes can also use serotonin to produce 5-hydroxy-indole-3-acetic-acid (5-HIAA) (85). Indoles and tryptamine are known AhR ligands (86–88) and thus function as immune modulatory compounds (89). For example, *Lactobacillus reuteri*-derived ILA activates AhR to generate immunoregulatory CD4^+^CD8^+^ intraepithelial lymphocytes (90). Further, the *CARD9* risk allele for inflammatory bowel disease (IBD) has been associated with reduced microbial indole production, and colonization with indole producing bacteria attenuates intestinal inflammation (91). Tryptamine and indole-3-acetate (I3A) both suppress pro-inflammatory responses in macrophages and hepatocytes (92). In addition, indole modulates incretin release from enteroendocrine cells and uncouples mitochondrial oxidative phosphorylation resulting in lower cellular ATP levels (93). It is therefore possible that indole could also suppress inflammation by regulating metabolism in immune cells. Moreover, indoles reinforce host intestinal barrier integrity (89) through pathways such as IL-22 production (94) or the pregnane X receptor (PXR) (95), both of which are imperative for maintaining intestinal homeostasis. Some bacteria also have the capacity to synthesize Kyn from Trp via the expression of IDO homologs (96–99). Alternatively, phosphoenolpyruvate and erythrose-4-phosphate are precursors for the shikimate pathway, by which microbes can synthesize aromatic amino acids, including Trp (100). Therefore, the microbiota has an immense potential to produce Trp metabolites, including Kyn, which have the capacity to modulate the host immune system (51, 52). Given the contribution of dysbiosis to autoimmunity, it is crucial to consider the microbiota as a source of skewed Trp metabolites and to evaluate the mechanisms by which they may contribute to autoimmune pathogenesis.

Proinflammatory cytokines, such as type 1 and type 2 interferons, upregulate IDO1 expression in DCs (101, 102). The resulting accumulation of Kyn increases Treg cell differentiation (103, 104) via the AhR pathway (104). AhR is a transcription factor activated by environmental pollutants in addition to a plethora of Trp metabolites, either derived from the microbiota and the endogenous Kyn pathway (105). Importantly, AhR activation by some of these ligands has been linked to the differentiation and function of both innate and adaptive immune cells (105), highlighting the importance of the Trp—AhR axis in modulating immunity. In addition, Trp depletion activates the stress kinase general control non-derepressible 2 (GCN2) due to the accumulation of uncharged tRNAs, which then induces cell cycle arrest and a state of anergy in effector T cells (106), leading to impaired proliferation and pro-inflammatory responses. Therefore, the Kyn pathway elicits immunosuppression by simultaneously inducing Treg cells and attenuating effector T cell responses.

### Tryptophan Metabolites in Autoimmunity

Although Kyn is considered an immunosuppressive metabolite, its exact role in autoimmunity is poorly understood. RA (39, 40, 43, 107, 108) and SLE patients (38, 41, 42, 44, 45, 48, 109) show a skewed distribution of Trp metabolites, characterized by an elevated Kyn/Trp ratio in the serum, urine, and PBMCs. Disease activity and clinical manifestations have been positively correlated with depleted Trp and increased Kyn (42, 44, 45, 48, 109) in SLE and RA (108). In addition, Kyn was one of the most increased metabolites in the PBMCs of SLE patients, and it was the best metabolite to discriminate between responders and non-responders to N-acetylcysteine treatment (45). The prevailing interpretation is that elevated levels of T1 IFN or other pro-inflammatory cytokines upregulate *IDO1* expression (109). An alternative non-exclusive hypothesis is that the dysbiotic microbiota may also have an enhanced capacity to metabolize Trp into Kyn or other metabolites.

Lupus-prone TC mice have serum metabolite alterations that mirror those of SLE patients, including an increased Kyn/Trp ratio (21). Additionally, compared to B6 controls, TC mice have less Trp and more Kyn in the serum regardless of the amount of dietary Trp consumed, suggesting an intrinsic skewing of Trp metabolism toward the Kyn pathway (21). Kyn serum concentration was positively correlated to autoantibody production in TC mice and increasing dietary Trp increased autoantibody and other autoimmune manifestations in this model, while a low Trp diet was protective (21). 1-methyl tryptophan (1-MT), an inhibitor of IDO1, had no effect on disease in TC mice (21), which may indicate a microbial origin from Kyn accumulation in this model. Alternatively, Kyn may accumulate because of a defect in downstream catabolic enzymes. Contrary to these results, 1-MT was therapeutic in MRL/*lpr* mice (110), suggesting that endogenous Trp metabolism may be more important in this model of lupus. Together, these studies highlight a pathogenic role for Trp metabolism and potentially Kyn itself in SLE.

Interestingly, Kyn activated mTOR in human PBMCs and the Jurkat T cell line (45). SLE CD4^+^ T cells possess a hyperactivated phenotype as well as signaling defects (111, 112). Cellular metabolism regulates T cell activation, proliferation, and differentiation (113–115), and SLE patient CD4^+^ T cells are characterized by increased mTOR activation and mitochondrial production of reactive oxygen species (116, 117). Rapamycin treatment normalizes T cell activation and decreases disease activity in SLE (118), demonstrating that enhanced mTOR activation contributes to disease. As a regulator of cellular metabolism, mTOR integrates cues from the environment such as nutrient and oxygen availability. Its activation allows for metabolic changes following T cell receptor stimulation to support proliferation and differentiation into effector T cell subsets (113–115, 119). Contrary to the established role of Kyn in immunosuppression, its accumulation in autoimmune patients as well as its activation of the mTOR pathway suggests that Kyn may be pro-inflammatory in lupus. In addition to microbial indoles, the plant-derived indole-3-carbinol (I3C) has been shown to promote wound healing in SLE patients by supporting M2-type macrophage reprogramming and promoting the expression of genes involved in the wound healing process (120). Thus, indoles may be globally beneficial in the context of autoimmunity.

Treg cells in RA patients showed a defective intrinsic induction of *IDO1* expression and Kyn synthesis for sustained suppressive capacity (121). Studies in murine models of RA also reported a defective Trp catabolism. 1-MT attenuated disease when given before disease onset (122). Additionally, 1-MT synergized with methotrexate, a common treatment for RA, to prevent autoimmune manifestations (122), highlighting a pathogenic role for Kyn synthesis in early phases of disease development in the K/BxN model. On the other hand, treatment with 1-MT or deletion of *Ido1* in CIA mice exacerbated disease severity by increasing IFNγ and IL-17 production (123). In the same study, administration of Kyn at disease onset prevented paw swelling, suggesting that Kyn is immunosuppressive in this model. *Ido2* deletion ameliorated disease in the K/BxN model (124), and direct targeting of IDO2 activity in B cells by administration of an IDO2-specific monoclonal antibody phenocopied *Ido2* deletion (125). In summary, blocking Kyn synthesis in spontaneous K/BxN mice is therapeutic, but it exacerbates disease in CIA mice. These opposing results may be due to differences in disease etiology, either spontaneous or induced in the context of acute inflammation. However, the use of 1-MT is controversial since differential inhibition of Trp catabolism depends on the 1-MT isomer used (126), and preferential inhibition of IDO2 is achieved with D-1-MT (79). This may explain inconsistencies between studies, underscoring a need for a more reliable inhibitor of this pathway. Collectively, these studies suggest that Trp endogenous enzymes, including IDO1 and IDO2, are important mediators of RA manifestations.

MS patients present a lower Kyn/Trp ratio in the urine (49) and cerebrospinal fluid (46), but a higher ratio in the serum (47), with a trend for reduced Trp and Kyn concentrations with disease progression (46). However, IDO expression was decreased in PBMCs of patients with stable disease compared to HCs, and was more drastically reduced along with the serum Kyn/Trp ratio in response to treatment (127). Moreover, a higher Kyn/Trp ratio was observed in patients with depression (46), likely due to decreased serotonin synthesis, and/or neuromodulatory effects of downstream kyurenines (80, 81), which are also perturbed in MS patients (47). Murine models of the disease have also implicated Trp metabolism. *Tdo2* deficiency (the gene encoding hepatic TDO) is protective in EAE (128). However, DC-targeted type 1 IFN treatment decreased EAE severity by upregulating *Ido1* expression and inducing a tolerogenic DC phenotype (129). Overall, these studies suggest that, contrary to SLE and RA, Trp metabolites may have an overall protective but complex effect in autoimmune CNS inflammation.

### Bacterial Trp Metabolites in Autoimmunity

While multiple studies reviewed above have implicated Trp metabolism in autoimmune diseases, they did not address the potential contribution of Trp metabolites of microbial origin. A synergy between type 1 IFNs and microbial Trp metabolites modulated astrocyte function to suppress inflammation in EAE (130). The protective role of Trp metabolites corresponded with reduced levels of indoles in the serum of MS patients (130). These microbial-derived Trp metabolites act as ligands for AhR through which they modulate the microglia-astrocyte crosstalk to reduce inflammation in MS (131). This suggests that the gut-brain axis could be targeted therapeutically in MS. Another contribution of microbial-mediated Trp catabolism in EAE was demonstrated in a study in which a Trp-restricted diet limited the expansion and function of autoreactive T cells (132). An interpretation of these seemingly opposite results may be that microbial-derived Trp metabolites have different modulating properties on immune responses in the microglia in the CNS compared to T cells in the periphery. Interestingly, bacterial-derived ILA and indole acetaldehyde were both decreased in the synovial fluid of RA patients (133), suggesting that loss of these Trp metabolites at the site of tissue injury may promote autoimmune pathology. In support of this hypothesis, the short chain fatty acid butyrate has been shown to increase the production of 5-HIAA from serotonin, which promotes Breg cell function through AhR and ameliorates murine autoimmune arthritis (85). Overall, these studies suggest that microbial Trp metabolites may play a significant role in MS and RA, although both pro-inflammatory and protective contributions have been reported.

Lupus-prone TC mice show the same pattern of elevated Kyn and reduced serotonin as SLE patients not only in the serum but also in their feces (21). An untargeted analysis of fecal metabolites identified Trp metabolism as one of the most differentially regulated pathways between TC and B6 mice (21). Broad-spectrum antibiotic treatment reduced fecal and serum Kyn levels in TC mice whereas it had no effect in B6 controls (21), strongly suggesting a microbial involvement in the skewing of Trp utilization in this model. Interestingly, Trp metabolism was also one of the pathways differentially represented in the feces of SLE patients (134). A direct contribution of Trp metabolites to autoimmune pathogenesis was demonstrated when high levels of dietary Trp exacerbated autoimmunity, whereas low dietary Trp was protective in TC mice (21). Furthermore, FMTs from TC mice consuming high dietary Trp induced immune activation in GF B6 recipients, whereas the microbiota from TC mice consuming low Trp did not (21). This suggested that the ability of the TC microbiota to induce immune activation depends on Trp metabolism.

A metabolic pathway analysis of 16S rDNA sequences suggested an increased microbial Trp degradation via a Kyn pathway in TC feces (21). In addition, the TC fecal microbiota is enriched in *Lactobacillus* and *Paraprevotella*, which have the capacity to catabolize Trp (21). TC mice consuming higher amounts of dietary Trp have even greater expansions of these bacteria, along with higher concentrations of Kyn relative to B6 (21), suggesting that this expansion may play a role in altered Trp metabolites. It is therefore likely that microbial Trp metabolites contribute to the inflammatory capacity of the TC microbiota, as there is no evidence for leaky gut or bacterial translocation in this model (21). The maintenance of intestinal barrier may be due to the expansion of *Lactobacillus* supporting barrier integrity through production of Trp metabolites (86). Together, these studies highlight a role for the microbiotal Trp metabolites in autoimmune pathogenesis. Further studies are necessary to identify these metabolites and the bacteria that produce them, as well as the mechanisms by which they promote autoimmunity.

## Conclusion

In summary, there is a strong body of evidence from clinical and mouse models associating microbial dysbiosis with autoimmune diseases. More studies in larger patient cohorts and across mouse models are necessary to define more precisely the changes in bacterial communities that are associated with disease, and how these changes occur relative to disease development. Only a small number of bacterial species have been identified to be responsible for specific autoimmune phenotypes. Some of these intestinal bacteria promote autoimmune disease by expressing genes with a high homology to genes in the mammalian host, further bolstering that molecular mimicry is a major mechanism of autoimmune activation. A major gap in the field is a mechanistic understanding of the bidirectional relationships between the development of dysbiosis and autoimmune pathogenesis. Investigations have and are continuing to reveal mechanisms by which gut resident microbes may contribute to autoimmune activation. However, special attention should be given to the ability of an altered microbiota to modify metabolites, such as those within the Trp pathway, since many have immune regulatory capabilities. Since there is clearly a relationship of altered Trp metabolism to autoimmunity, future studies should investigate how these metabolites, including Kyn, could be pro-inflammatory in this context. Growing evidence challenges the well-established notion that Kyn is an immunosuppressive metabolite and highlights the need for a more detailed understanding of immune-modulatory metabolites in various disease contexts. Both pro-inflammatory and anti-inflammatory effects of specific groups of bacteria are observed in different models of the same disease, such as the effects of *Lactobacillus* in lupus mouse models, further emphasizing a need to understand specific mechanisms by which the microbiota regulates autoimmunity. Finally, information on the timing of microbiota contributions to disease is scarce, and thus, should be an important question to address in the future.

## Author Contributions

JB, BR, and LM wrote the review. All authors contributed to the article and approved the submitted version.

## Conflict of Interest

The authors declare that the research was conducted in the absence of any commercial or financial relationships that could be construed as a potential conflict of interest.
